# Web-Based System for the Remote Monitoring and Management of Precision Irrigation: A Case Study in an Arid Region of Argentina

**DOI:** 10.3390/s18113847

**Published:** 2018-11-09

**Authors:** Flavio Capraro, Santiago Tosetti, Francisco Rossomando, Vicente Mut, Facundo Vita Serman

**Affiliations:** 1 Instituto de Automática (INAUT), UNSJ–CONICET, Av. Lib. Gral. San Martín 1109 (oeste), San Juan J5400ARL, Argentina; stosetti@inaut.unsj.edu.ar (S.T.); frosoma@inaut.unsj.edu.ar (F.R.); vmut@inaut.unsj.edu.ar (V.M.); 2 Instituto Nacional de Tecnología Agropecuaria (INTA), E.E.A. San Juan. Ing. Marcos Zalazar (Calle 11) y Vidart, Pocito, San Juan J5429XAB, Argentina; vita.facundo@inta.gob.ar

**Keywords:** remote monitoring, precision irrigation, automatic control, soil moisture, drip irrigation, sensors

## Abstract

This article presents a description of the design, development, and implementation of web-based software and dedicated hardware which allows for the remote monitoring and control of a drip irrigation system. The hardware consists of in-field stations which are strategically distributed in the field and equipped with different sensors and communication devices; a weather station and drip irrigation system complete the setup. The web-based software makes it possible to remotely access and process the information gathered by all the stations and the irrigation controller. The proposed system was implemented in a young olive orchard, located in the province of San Juan, an arid region of Argentina. The system was installed and evaluated during the seasons 2014–2015 and 2015–2016. Four regulated irrigation strategies were proposed in the olive orchard to test its behavior. In this pilot experiment, the precision irrigation system was a useful tool for precisely managing the irrigation process, applying only the required amount of water (precise irrigation). Regulated deficit irrigation experiments, on the other hand, have demonstrated the sensitivity of olives to water restriction. The precision irrigation system made it possible to control soil moisture levels, avoiding water stress in the control treatment.

## 1. Introduction

Over the last two decades, agriculture systems have benefited from the incorporation of technological advances developed for other industries, such as GPS, communication systems, and imaging systems [1]. Traditional agriculture was initially enhanced by the introduction of machines and the use of synthesized fertilizers [2]. Later on, advances came from genetics and the automation of agricultural processes. Recently, the age of communications and information technologies (IT) has allowed for the integration of new devices, methods, and procedures to develop precision agriculture (PA) [3]. Nowadays, the use of sensors, intelligent actuators, onboard computers, communication systems, information processing, remote control and monitoring, and global positioning systems (GPS) is becoming a must in modern agriculture.

In the context of PA, temporal and spatial soil factors and crop development are relevant topics that are widely documented in the literature [4,5,6]. Before the introduction of agricultural machines, production units used to be small, and producers were able to manually accomplish the different tasks related to the crops. However, with the intensive use of machines, the size of the production units increased, and the analysis and interpretation of field data became difficult without the use of new technologies [7].

### 1.1. Precision Agriculture in Argentina

In Argentina, the inclusion of new technologies in agricultural processes has been more noticeable in extensive agriculture (e.g., soybeans, corn, wheat, and sunflower), where computer-guided machinery, automated planting, and selective fertilization, among others technologies, have been applied [8].

However, in the region of the Andean Valleys, and particularly in the provinces of San Juan, Mendoza, La Rioja, and Catamarca (an area with a desert and semi-desert climate), new technologies are still required to meet the needs of the local crops [9,10], which in this area are mostly oriented to fruits trees (olives and vineyards) and vegetables (tomato, onions, and garlic, among others). In these provinces, technologies related to irrigation management and the determination of crop water requirements are critical.

Water use efficiency and availability are critical factors in this region. A low rainfall level, ranging from 87 mm per year in the province of San Juan (Tulum Valley) to 415 mm per year in La Rioja (capital city), is usual in this area. Precipitations are concentrated in the period November–March, although the ambient relative humidity (RH) is usually very low all year long (RH<40%). High average temperatures and low average relative humidity result in high reference evapotranspiration ($ET_{0}$), with consumption levels of up to 1500 mm per year in the Tulum Valley of San Juan [10], conditioning the type of crops adapted to this climate. Particularly, most olive tree varieties are well-adapted to low water use rates [11].

In this region, water comes from melting in the high mountain and accumulates in dams and underground layers. Then, it is obtained from deep water tables and distributed through irrigation systems, hence the importance of monitoring water use and controlling the irrigation equipment to implement efficient irrigation schedules, both spatially and temporally.

In addition to the climate characteristics of the region, other factors, such as population growth and the development of other production activities (industry and mining), are causing water scarcity for irrigation, which is becoming the most limiting factor for the development of fruits and horticultural activities.

For these reasons, farmers need technologies aimed at monitoring and optimizing the use of water, fertilizers, and electric energy [12].

### 1.2. Traditional Irrigation and Precision Irrigation

The irrigation process is defined as “the artificial application of water to soil for the purpose of supplying the water essential to plant growth” [13] with three objectives: (i) to compensate for deficiencies of moisture in the soil; (ii) to improve environmental conditions of the soil and crop; and (iii) to apply nutrients and plant protectors [14].

Irrigation requires proper scheduling to be effective. Irrigation scheduling is a set of technical procedures for deciding when and how much water to apply to a particular crop or irrigated zone. The first question relates to defining irrigation frequency, which is the time between the start of two consecutive water applications. The second question addresses the problem of determining the amount of water to be applied in each irrigation event. In general, irrigation scheduling is based on the crop water requirement ($ET_{c}$), obtained from the reference evapotranspiration ($ET_{0}$) calculation and the crop coefficient ($K_{c}$) [15]. The measurement of the soil water content [9,16,17] or plant water status [18,19,20,21] provides additional information to define an efficient irrigation schedule.

In traditional irrigation management, farmers still apply the concept of “uniform irrigation”, which involves applying water uniformly over every part of the field, without considering the spatial variability of soil properties and crop water needs, causing over-irrigation in some parts of the field while other parts of the field are under-irrigated [22].

On the other hand, Precision Irrigation (PI) proposes designing irrigation systems to achieve “differentiated irrigation units”, taking into account the spatial soil variation, different phenological requirements, and water availability in the area [23].

Precision Irrigation is defined as “the accurate and precise application of water to meet the specific requirements of individual management units or plants and minimize the adverse environmental impact” [24,25].

The concept proposes a new approach to irrigation management from the perspective of control systems. Crop yields are optimized through: the systematic gathering of data; the analysis of information from the weather, soil, and crop; efficient irrigation management and the use of technology for its implementation; online fault detection; modeling of the soil-water-plant system; and the application of different control techniques. The PI concept can be summarized as “an irrigation system that knows what to do, knows how to do it, knows what it has done, and learns from what it has done” [26].

### 1.3. Local Situation and Objectives

In the arid regions of Argentina, the use of PI is essential for the development of sustainable agriculture, in a future scenario of water scarcity due to a growing population and industry that compete for hydric resources. The benefits of PI can also be maximized by incorporating additional technologies into agriculture [23,26]. Under this paradigm, farmers are replacing traditional irrigation methods (surface or furrow) with modern pressurized irrigation systems such as drip irrigation, micro-sprinklers, or subsurface systems, in order to apply water only to the root zone of the crop. The use of new technologies should be complemented with careful irrigation scheduling.

A review of local olive producers’ practices shows that most of the pressurized irrigation systems installed are manually controlled. That is, irrigation scheduling is usually performed empirically by the farmer, by estimating needs based on the historical $ET_{0}$, or in the best case by calculating the current consumption (monthly or annual $ET_{c}$) based on data provided by agro-meteorological stations located in the area. In extensive areas, irrigation system performance is difficult to evaluate through visual inspection. Any failure or problem can take several days to be detected and solved, depending on the problem presented. Irrigation problems are then detected when the crops present water stress symptoms.

In recent works, the integration of new technologies into sensor networks and field measurement stations [27,28,29] has proven to be very useful for irrigation management, combining tasks of monitoring and control to optimize water use [30,31,32,33].

This paper presents the development and implementation of a precision irrigation system. The system consists of web-based software that gives the user access to the information from different measurement stations specially designed to be installed in the field and to control irrigation equipment and irrigated zones as necessary. The system can be used in complement with any irrigation technology (even with surface or furrow irrigation), crop type, and scale of production.

The system has been designed to provide agronomists, producers, advisors, and owners that are located far from the orchard with real-time information about the variables of the crop, the weather, and the irrigation system, in order to improve the decision-making process, alleviate weather effects, or try different irrigation treatments, while avoiding traveling long distances, and saving time and fuel.

Even though the system was designed with the region’s climatic conditions in mind, there are other places around the world where climatic or economic conditions require precise monitoring and control of the irrigation process.

Climate change is modifying the rainfall regime around the world, and places where no irrigation was necessary now require supplemental irrigation. In this case, producers may need to control and monitor the irrigation process, given the high costs related to energy and water fees. On the other hand, in water scarcity areas, water, not land, is in general the most limiting resource. Under such conditions, it seems natural to maximize the return per unit of water rather than maximize the return per unit of land. Water productivity can be significantly improved by applying irrigation monitoring and control technologies in areas with supplemental irrigation as well as in areas with full irrigation.

To the best of the authors’ knowledge, there are no systems like the one presented in this work installed in the region. Although systems with some similarities to the one presented are installed in Spain, Israel and, Australia, most of them are based on central pivots or linear systems with sprinklers, and in general, they only control irrigation but do not include information about $ET_{c}$ [26,28,34,35,36,37]. In recent years, some commercial systems, such as Bee2Crop, NetBeat of Netafim, AgSense ICON of Valley NetBeat, and others have become available, but they are expensive for a small or medium farmer and are sold as closed solutions. In addition, support and maintenance are complex, due to the proprietary hardware and software. The system presented in this article was designed with standard industrial grade components, which is why it constitutes a new technological tool for local producers and scientists.

## 2. Materials and Methods

### 2.1. Description of the Developed Precision Irrigation System (PISys)

Precision Irrigation System (PISys) is a tool, developed by the authors at the Institute of Automation that combines web-based software and an arrangement of different sensors, actuators, and communication devices. All the components work together to collect, process, and present all the information related to the performance of the irrigation process to local and remote users.

The software allows for real-time monitoring of different variables, such as soil moisture at different depths, weather variables, pressure and flow in the main irrigation line, and valves and pump status (open/closed—on/off). Sensors are installed in the measurement stations, located at specific sites on the orchard and the irrigation equipment.

The use of the proposed system allows for precision irrigation of each irrigation unit, by adjusting the amount of water applied as a function of the soil moisture and the $ET_{c}$.

**Figure 1:**
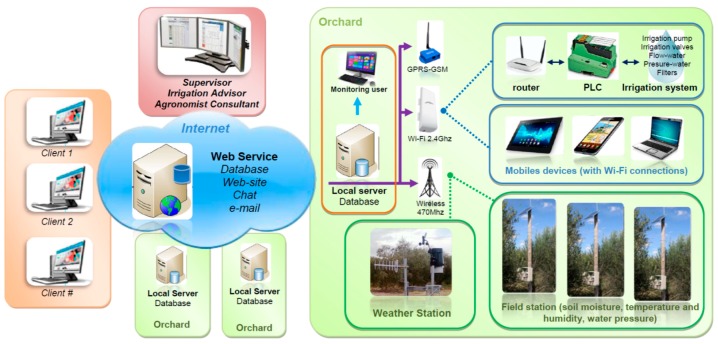
General view of the system developed. The principal components are: in-field devices, the web server, remote clients, and the supervisor.

Figure 1 presents an overview of the elements of the PISys, which are described in the following lines.

#### 2.1.1. In-Field Devices

Figure 1 displays all the devices installed in the orchard. These elements are marked with a green frame.

The Local Server is a Personal Computer (PC) that can also be a user terminal, where the irrigation system and variables can be monitored.

The Local Server PC runs a program that makes it possible to gather the information from several measurement stations, from automatic weather stations, and from the controller system installed at the irrigation station. Information is then processed and stored in a local database. If necessary, the user can locally access the information in the form of raw data or graphics.

The system is configured so that all the stations perform a measurement every ten minutes. The measurements of all the stations are synchronized and stored locally. Every hour, the system downloads the information from all the stations to the server. The user can configure the frequency of this process. 

These tasks do not require high computational requirements, but it is necessary to use a robust PC and an uninterruptible power system to assure continuous service.

The Local Server has four communication links, each with a specific use:

- An Internet connection makes it possible to transmit data to a Web Server. This connection is also used to access the system remotely.
- A Wi-Fi network (Ubiquity Nanostation M2 2.4 GHz) links a Programmable Logic Controller (PLC), installed at the irrigation system, with the server PC and allows for the use of mobile devices, such as tablets or smartphones, to locally connect to the server.
- A Radio Modem device, at 470 MHz, is used to communicate with the measurement stations and the weather stations.
- A mobile phone connection (GPRS-GSM modem, G24 Motorola) allows the system to send text messages with alarms or other information related to the system functioning. In the event of a failure in the internet connection, this system act as an auxiliary link to access the information on the server PC.

**Figure 2:**
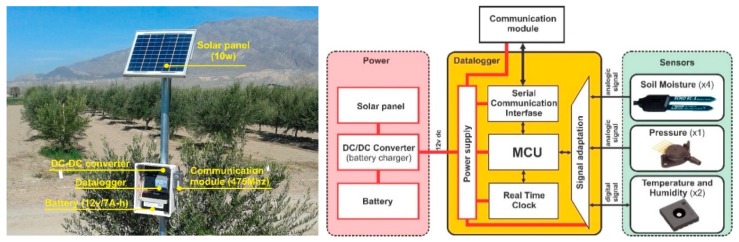
Components of an in-field measurement station.

The Field Stations consist of a data logger, a radio device for wireless data transmission, a power source (solar panel, charger, and battery), and one or more sensors. Figure 2 presents a schematic diagram of the field station.

The data logger used in the PISys has been designed and developed by the authors at the Institute of Automation, National University of San Juan. The device (named Agromet) is based on a low power consumption microcontroller unit (MCU) MC9S08QE32, 8 bit, from NXP Semiconductors. The datalogger periodically reads the value of the sensors connected to the device, analyzes failures in the station, processes the information from the sensors, controls the communication node, and sends the information stored in the memory to the server.

Each field station is equipped with a communication module that allows the measuring stations to be part of a wireless data network, so that nodes on the same network and the PC server can communicate with each other. To cover long distances between nodes, and to alleviate the effect of signal attenuation due to vegetation being more pronounced in fruit crops [38,39] such as olive trees, wireless networks operating in low-frequency bands should be used [40]. Long-range and low-consumption radios from Appcom Technologies, APC230N (450 Mhz) are used in PISys; the modules are equipped with 3 dBi omnidirectional antennas to cover the distances between the nodes up to 1500 m and with 12 dBi five-element Yagi antennas to reach distances between the nodes up to 4000 m.

Measuring stations have four soil moisture sensors, one ambient relative humidity and temperature sensor (model SHT31 from Sensirion, Staefa, Switzerland), and one pressure sensor (model MPX5700GP, NXP, Eindhoven, The Netherlands) installed in an irrigation lateral (Figure 2). The capacitive-type soil moisture sensors are from Decagon, model EC-5 (Meter Environment, Pullman, WA, USA).

Based on previous experiments, soil moisture sensors are installed at different depths. One is installed at the highest root density (0.3 m) to evaluate the dynamic changes in the soil water content. Two more sensors are installed deeper, but still within the root zone (0.6 m and 0.9 m); and finally, one more sensor is installed below the root zone (1.10 m), to detect any water leakage or the presence of a water table. Sensors for ambient air humidity and temperature are installed at 0.5 m and 1.50 m to measure the thermal gradient and evaluate the effects of frost or high temperatures. These sensors also make it possible to calculate the local thermal sum (growing degree-days and chill hours). A pressure sensor is inserted into the irrigation line to monitor the line pressure.

Each in-field station is powered by a 10 W solar panel, a voltage regulator, and a 4 Ah rechargeable sealed lead acid battery that provides approximately four months of autonomy.

Weather stations (Vantage-Pro2, from Davis Instruments, Hayward, CA, USA) are equipped with ambient temperature and relative humidity, solar radiation, ultraviolet light sensor, atmospheric pressure, wind speed and direction, and rainfall sensors. A wireless communication module links the station to the server to transmit the meteorological variables. A solar panel power system completes the installation. The weather station was adequately located and installed following the requirements for site preparation and sensor placing [41]. Hourly reference crop evapotranspiration, $ET_{o}$, is estimated using the Penman-Monteith equation (FAO-56 model) [15].

In the Irrigation Station, the principal control element is a Programmable Logic Controller (PLC) (model ILC150-ETH from Phoenix Contact, Blomberg, Germany). The PLC is responsible for controlling the irrigation, according to the schedule defined by the user. The PLC controls the water pump and the sub-main valves for each irrigation unit. At the same time, the PLC measures the pressure and flow in the main irrigation pipe, the pressure in the filter system, the electric power consumption, the amount of fertilizer incorporated into the irrigation line, and the status (on/off) of the pump and irrigation valves. All this information is sent to the server PC using the Ethernet port of the PLC and a Wi-Fi router. A 2000W uninterruptible power supply is also installed to protect the system.

#### 2.1.2. Web Service

The Web Service is provided by a hosting service (blue cloud in Figure 1); it acts as a backup and allows quick access for remote users (operators, managers, or external consultants). The users can find all the information regarding the irrigation process or the weather. A username and password should be provided to access the specific information of each production site or orchard.

#### 2.1.3. Clients and Remote Users

Clients and remote users (marked in orange in Figure 1) can access the information from any point outside of the orchard. Any device connected to the internet with a web browser should be able to connect to the system. Remote users are growers or managers that are located far from the orchard and need to control the irrigation equipment and to monitor the variables of the crop.

#### 2.1.4. External Supervisor

The external supervisor (red box in Figure 1) plays an essential role in the use of the PISys. The supervisor can visualize the status of one or more orchards in the web server. A supervisor can be an irrigation specialist, an agronomist, an agricultural consulting, a field manager, or even a working group formed of different specialists.

### 2.2. Web-Based Software

A web application was developed to ensure secure and real-time access to the information.

The back-end of the system is programmed in the PHP Hypertext Pre-Processor [42]. On the other hand, the front-end that interacts with the user is programmed in Javascript [43] to generate Dynamic HTML. The data are stored and managed using a database in MySQL [44].

As mentioned above, the system has more than one server. The local server is connected to a Local Area Network that handles the data coming from in-field stations. Information on this server can be accessed via a web browser. Since one of the main features of the system is that the information can be accessed from different places, a challenge in the implementation was to select the best option for synchronizing information between the local server and the remote or external server. A limiting factor in this sense was the fact that there was not a good internet service in the location where the system was installed. In this case, a GPRS modem was used to synchronize the servers.

Python [45] was used to program the interface between the local (in-field) and the remote server. This language was also used on the local server to process the information from the weather station, the measurement stations, and the irrigation station.

Information at the web server is synchronized every 10 min, except for information related to the irrigation system operation, which is updated more frequently, between 3 s and 10 s, depending on the internet connection speed.

Once the user logs onto the system, the PISys information is displayed in the form of a horizontal tabbed Graphic User Interface (GUI) with seven different tabs to allow for navigation within the different information and configuration pages.

**Figure 3:**
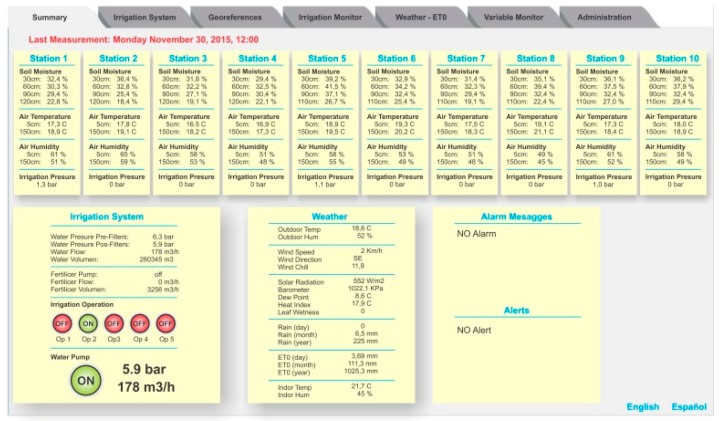
The Summary tab shows all the updated process information.

The Summary tab (Figure 3) is the first tab displayed. This tab presents the main variables and parameters from each measurement station. This tab also contains different alarms and messages related to the irrigation system operation and measurement stations status. In this way, the general state of the overall process is presented to the user at a glance. The language can be switched between English and Spanish.

**Figure 4:**
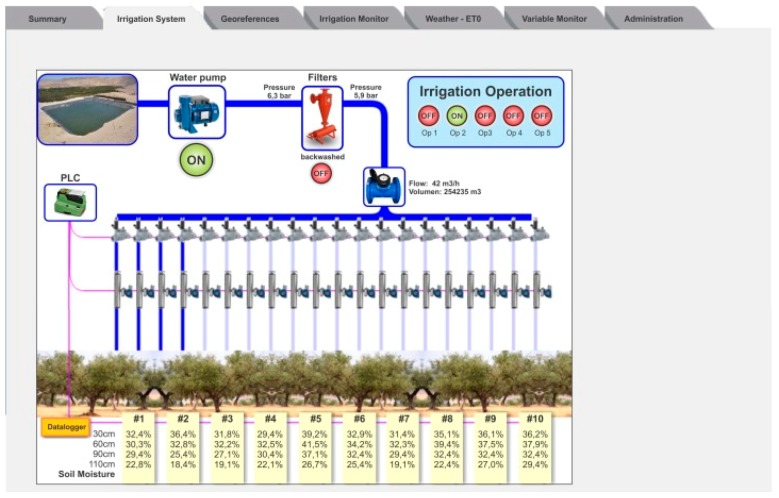
The Irrigation System tab shows information related to the irrigation system operation.

The Irrigation System tab allows the user to monitor and control the irrigation system (Figure 4). Measurements of pressure before and after the filters, flow in the main pipe, and the status of the irrigation pump and valves are presented. In this area, it is also possible to set the irrigation scheduling to be executed automatically, select the fertilizer dose, and program the filter backwashing. Buttons for manual operation of the system are also found here.

**Figure 5:**
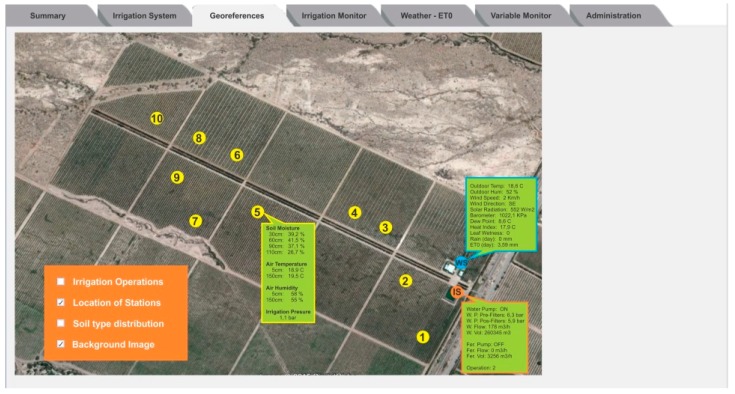
The Georeference tab presents information from each measurement station.

Figure 5 shows the content of the Georeference tab. This tab presents information from the PISys projected over an aerial photo of the field. Different information layers (watering shifts, location of each in-field station, soil type distribution) can be activated. In this screen, it is also possible to see the last values acquired from the stations, as the mouse passes over the location points in the map.

**Figure 6:**
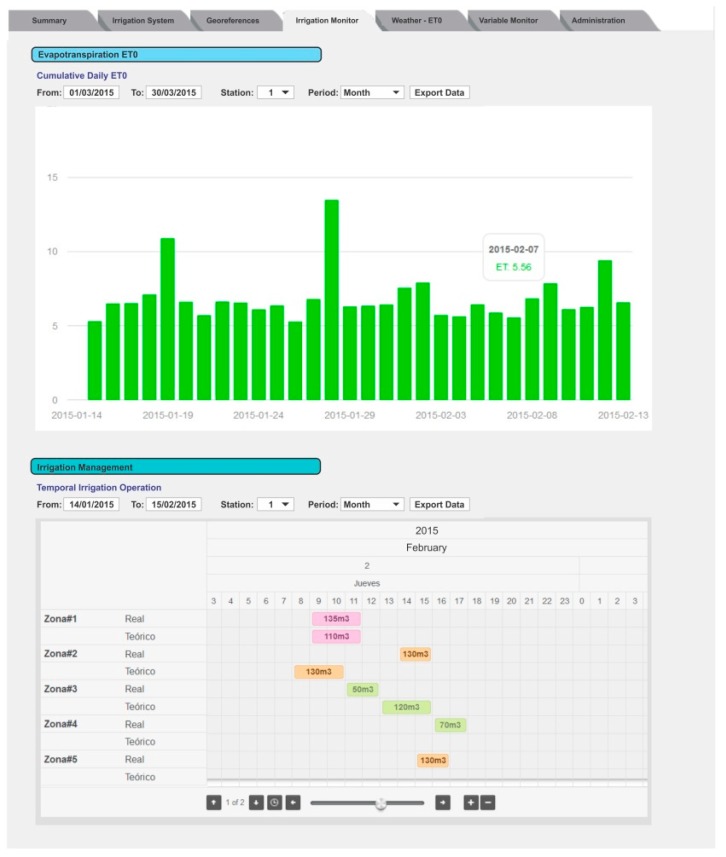
The Irrigation Monitor tab shows information related to the irrigation programming. The cumulative daily $ET_{o}$ and the irrigation shifts are also presented in this tab.

In the Irrigation Monitor tab, Figure 6, two graphics related to watering are available. At the top of the tab, a bar graph shows the cumulative daily $ET_{0}$. If the user enters the $K_{c}$, the system can also present the $ET_{c}$ values. At the bottom, another graphic shows the watering schedule (when and how much water to apply), and the amount of water that was applied effectively. In the event of a difference between these values, the program can issue an alarm to the user.

The remote client can use this information to determine the irrigation scheduling, and then introduce it into the program.

Information is presented in the form of temporal diagrams, allowing the user to analyze the water balance for each irrigation unit and, if necessary, adjust the irrigation schedule.

**Figure 7:**
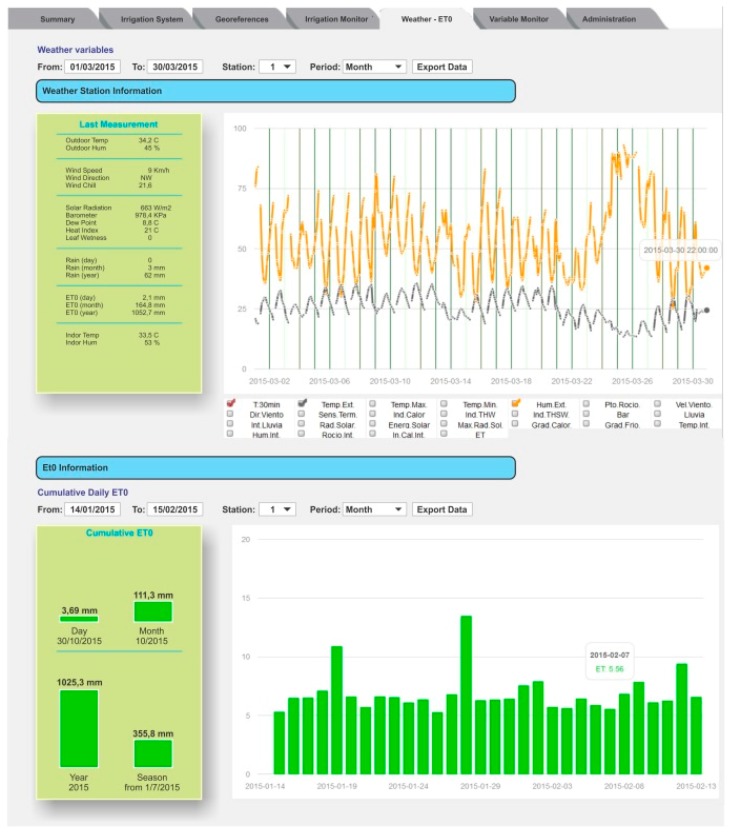
The Weather–$ET_{0}$ tab displays the evolution of the weather variables and the cumulative daily $ET_{o}$ estimation.

Figure 7 shows the content of the Weather tab. This tab also contains two graphics. In the upper graph, the user can explore all the information related to the weather by selecting the variables and the period to show. The lower graph presents another view of the daily $ET_{o}$ where the user can select the period to show.

**Figure 8:**
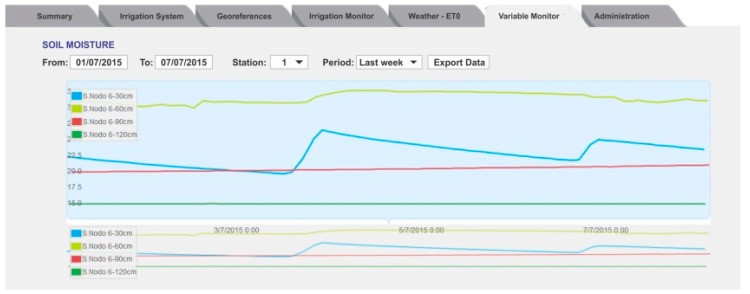
The Variables Monitor tab shows the soil moisture levels in a given period.

In the Variables Monitor tab, the moisture level for each station can be plotted (Figure 8). The user can select the measurement station, the sensor in the station, and the period to show the behavior of the soil moisture at that point.

**Figure 9:**
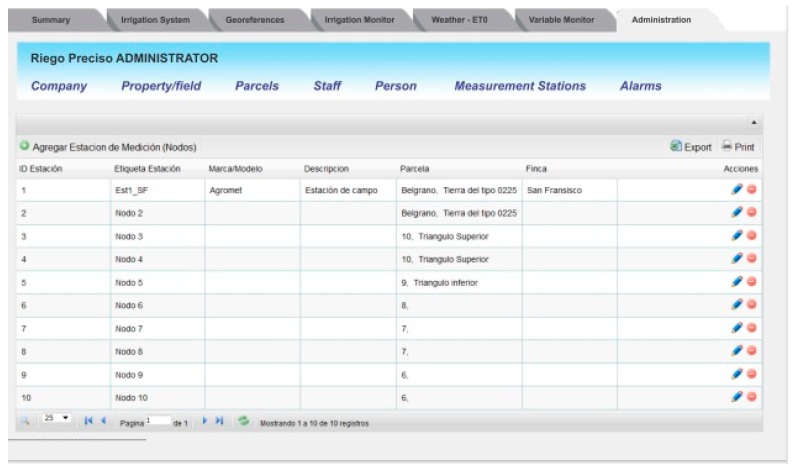
The Administration tab allows the system administrator to configure different parameters of the monitoring and control system.

Figure 9 presents the content of the Administration tab. This tab allows the user to set different parameters related to the way the information is presented, the frequency of synchronization among the servers, the sampling time for in-field stations, the labeling of sensors, the actuators and communication nodes, and entering GPS location information for each station, in-field station configuration, among other parameters. This tab can be accessed only by those users with administrator attributes.

## 3. Field Implementation

### 3.1. Description of the Orchard

The province of San Juan, Argentina, has a cultivated extension of about 110,000 Ha, with vineyards (50%) and olives (20%) being the main crops. Better global market conditions for olive oil have generated an increasing interest in this crop [11].

**Figure 10:**
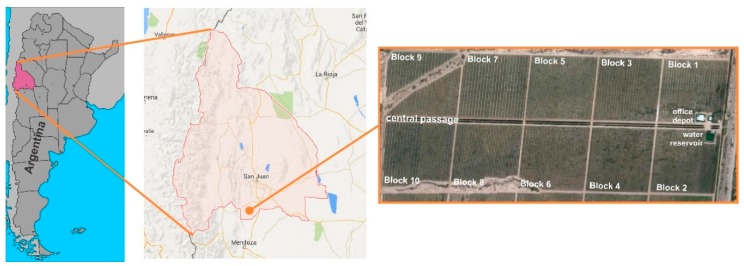
Location of the experimental olive orchard, in Cañada Honda, province San Juan.

The PISys was installed in a commercial farm, with an intensive olive orchard (ca. ten years old), of about 90 Ha. The orchard is located in Cañada Honda, south of San Juan, (32°2′ S, 68°32′ W, 620 m elevation), a relatively flat area at the foot of the Andes, characterized by a desert climate with a high temperature in summer and low rainfall. Figure 10 presents an aerial photograph of the olive orchard, which is divided into ten irrigation blocks of about 9 Ha each. Table 1 contains details of the olive orchard and the hydraulic parameters of the drip irrigation system.

### 3.2. Irrigation Equipment

**Figure 11:**
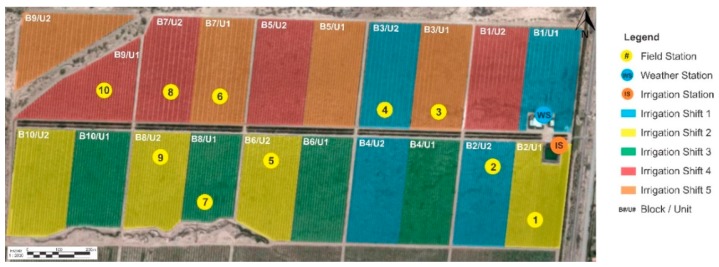
Distribution of twenty irrigation units, arranged in five irrigation shifts (marked in colors). The location of the measurement stations is indicated with circles.

The irrigation station is located on block number 2 (Figure 11). From this point, the main pipe runs parallel to the main alley, for about 1200 m.

Each orchard block is divided into two irrigation units; therefore, the irrigation network has twenty irrigation units, each with an irrigation valve. Due to the limited pump capacity, it is only possible to activate four irrigating valves at a time, resulting in a sequence of five irrigation shifts. Table 1 summarizes the hydraulic configuration. Figure 11 presents the distribution of the irrigation units, identified with different colors according to the irrigation shifts. Two drip lines (with 3.5 L/h in-line compensating drippers) are installed for each plantation row. The main pump is 150 HP, and the section of the main pipe is 0.50 m. The irrigation system was designed to apply an irrigation precipitation rate (IPR) of 1.17 mm/h or 1.2 mm/h, depending on the orchard variety and plant spacing, according to Table 1. IPR is the rate at which the drip irrigation system applies water in one hour.

### 3.3. In-Field Measurement Stations

Ten measurement stations were installed on different irrigation units, as shown in Figure 11. The weather variables were obtained from a weather station located near the orchard.

**Figure 12:**
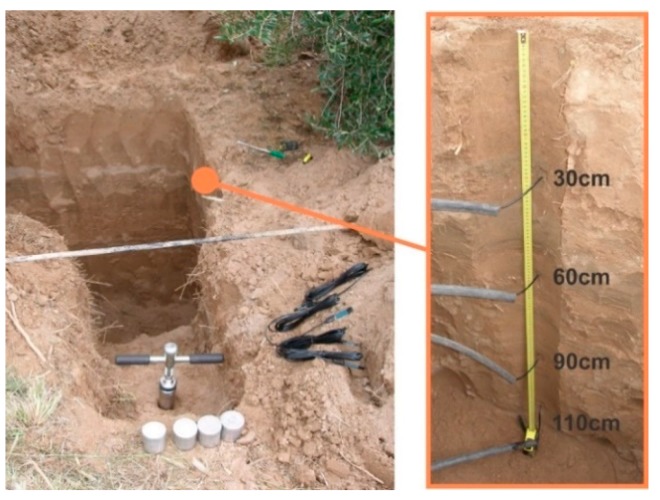
Soil pit performed to install four soil moisture sensors, model EC-5 from Decagon. Ten soil pits like this were made to install sensors and extract soil samples.

The locations of the measurement stations were defined considering irrigation shifts, olive variety, and soil type profile. Ten soil pits were made to install the soil moisture sensors (Figure 12). This procedure made it possible to estimate the root depth and distribution, and to install the soil moisture sensors in suitable places. In most cases, the higher root density was found in the first 0.3 m depth, and the roots were no deeper than 1 m. A soil sample of 100 cm^3^ was taken, close to each sensor, to determine the dry bulk density and volumetric moisture. At the same time, a soil sample of about 3000 cm^3^ was taken to perform the gravimetric moisture laboratory calibration of each sensor, following the method proposed in Reference [46]. Moreover, once every 20 days a soil sample from the same depth as the sensors was taken, obtained using a soil auger, to evaluate the soil moisture and recalibrate the sensors if necessary.

### 3.4. Irrigation Strategies

During the season 2015–2016, a full irrigation strategy was proposed, in order to replenish, in every application, the amount of water consumed by the crop (100% of $ET_{c}$), considering

(1) $$\;ET_{c}=ET_{0}\times K_{c}\;$$

The $ET_{c}$ was calculated applying a crop coefficient of $K_{c}=0.55$, as defined by the FAO [15] and revised for olive orchards by Orgaz et al. [47]. Given the climatic conditions in the area (arid, without winter rainfalls), the value of $K_{c}$ can be kept constant throughout the whole season, as is proposed in Reference [48].

An interesting irrigation strategy is to supply water below the crop requirements, causing a temporary water deficit in a specific phenological stage [49]. This strategy, known as Regulated Deficit Irrigation (RDI), is commonly used with some fruit trees in order to reduce the amount of water applied with minimal or no reductions in fruit production [50]. For olive trees, the second phase of fruit growth, corresponding to the pit-hardening period, is defined as the period of least sensitivity and highest resistance to water deficit [51,52].

**Figure 13:**
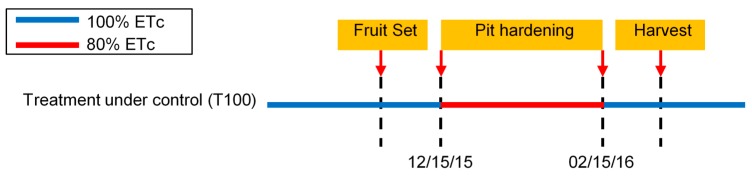
Timestamp diagram for the application of the RDI strategy in the course of the pit-hardening period in cv. *Arbequina*.

A pilot monitoring experiment is then proposed, where an RDI treatment is applied during the pit-hardening period on the *Arbequina* variety (block 3, irrigation unit 1, station 3, B3/U1-S3). The pit-hardening phenological period goes from 15 December to 15 February, and in the course of this stage, ${\mathrm{ET}}_{c}$ is calculated as:

(2) $$ET_{c}=ET_{0}\times K_{c}\times K_{cj},$$

where $K_{c}=0.55$, and $K_{cj}=0.8$ is applied only during the pit-hardening period, while the rest of the time $K_{cj}=1$, Figure 13. This treatment was labeled as T100 and was under PISys monitoring.

### 3.5. Irrigation Treatments

In addition to the RDI strategy applied in B3/U1-S3 (T100), a Sustained Deficit Irrigation (SDI) treatment was also implemented in some parts of B3/U1. SDI consisted in reducing the amount of irrigation applied during the whole season. The treatments tested were 80% (T80), 60% (T60), and 40% (T40) of the amount of water applied to the treatment under PISys control (T100), and were applied to some reduced areas of B3/U1. These irrigation treatments are out of the scope of the monitoring experiment, but the results are useful to evaluate the behavior of the system monitoring T100.

The treatments (T80, T60 y T40) consisted in proportionally reducing the IPR by reducing the number of drippers in the drip line of each treatment after fulfilling the soil profile. This configuration was maintained throughout the season.

The Stem Water Potential (SWP) $\Psi_{x}$ of treatments T100, T80, T60 and T40 was periodically measured for control, as is proposed by Reference [53].

### 3.6. Irrigation Programming Set

Depending on the irrigation strategy, PISys helps the user to estimate the $ET_{c}$ (Equation (2)). The user must enter the $K_{c}$ values in the Irrigation Monitor tab (Figure 6). In the event that $K_{c}$ varies throughout the season, the user must also enter the period corresponding to each $K_{c}$. The system uses this information to calculate the difference between the theoretical water application and the water effectively applied.

At the beginning of the growing season, it must be ascertained whether the soil water profile is partially or entirely filled. Partial filling makes it possible to take advantage of the occasional rainfall. In the case of this experiment, it was decided to fully fill the root zone, given the low rainfall rate in the area and the increasing water demand of the crop.

Only effective rainfalls (>9 mm) should be included in the calculation of the irrigation cycle. The number of irrigation shifts determines the watering frequency. Also, the irrigation system is not able to water all the crop at the same time, so irrigation priorities need to be established, minimum and maximum soil moisture levels defined (Figure 9), and all irrigation system restrictions considered (Table 1).

With this information, the system calculates the irrigation as:

(3) $$\;Irr\left[\mathrm{mm}\right]=\left(ET_{0Accum}\left[\mathrm{mm}\right]\times K_{c}\times K_{cj}\right)-Rain_{Effec}\;\left[\mathrm{mm}\right]\;$$

(4) $$\;Irr_{ON}\left[h\right]=\frac{Irr\left[\mathrm{mm}\right]}{IPR\left[\mathrm{mm}/h\right]}\;$$

where Irr is the amount of water to replenish, $ET_{0Accum}$ is the cumulated $ET_{0}$ since the last irrigation, $K_{c}$ and $K_{cj}$ are the crop coefficients, $Rain_{Effec}$ is the effective rain, $Irr_{ON}$ is the duration of the irrigation, and *IPR* is the irrigation precipitation rate of the system.

In the proposed experiment,$\;R_{T100}=1.17\;\mathrm{mm}/h$, $IPR_{T80}=0.936\;\mathrm{mm}/h$, $IPR_{T60}=0.702\;\mathrm{mm}/h$, and $IPR_{T40}=0.468\;\mathrm{mm}/h$.

Once the water requirements and irrigation programming have been calculated, watering is managed by PISys. The user introduces the irrigation schedule into the web-based software, and the irrigation program is then executed by the PLC installed at the irrigation station.

## 4. Results and Discussion

### 4.1. Watering Monitoring

The system provides the user with actual information related to flow and soil moisture, as well as the zones where watering is active. The user can also observe and control the status of each element of the irrigation system, and see the time evolution of the soil moisture at the measurement stations. The PISys can also signal any failure or abnormal behavior of the system.

The use of PISys eases information analysis. The user can learn from previous irrigation experiments and improve the irrigation strategies.

The remote web access also makes it possible to share experiences with other users. The main advantage of the proposed system is that every person involved in the crop process can access all the information (depending on the security level assigned). This characteristic helps ensure fast interaction among field personnel, managers (generally located in the city), agronomical consultants, and even some suppliers. The analysis of the different events can lead to taking better decisions, reducing risks, and increasing field productivity.

The role of the external supervisor is well-known in the agronomic field, but it is hard to find growers that request the services of an irrigation specialist. One of the objectives of the development of the PISys is to provide a technological tool for these specialists, giving them as much information as possible about the processes that are carried out in the farm or field. This tool helps advise farmers quickly and objectively about orchard management, efficient irrigation equipment management, and resource optimization.

### 4.2. Pilot Experiences Results

**Figure 14:**
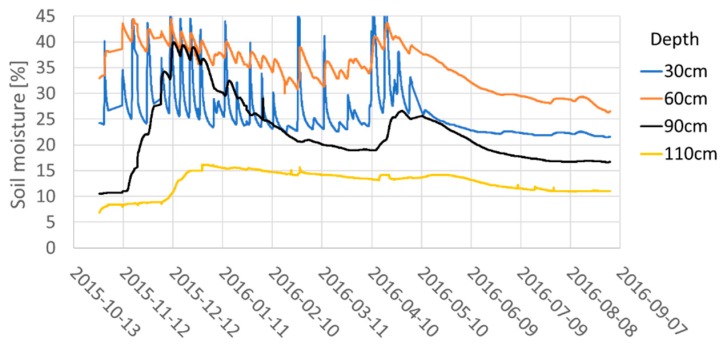
Soil moisture measurements of Station 3, at Block 3, Unit 1. Throughout the pit-hardening period (12/15/2015–02/15/2016), an RDI was applied. This effect is more noticeable at the 0.9 m depth sensor.

The performance of the PISys at the level of one irrigation unit is shown in Figure 14, where the soil moisture in field station 3 at B3/U1 is presented. The effect of the RDI treatment, applied during the pit-hardening period, can be appreciated, mainly in the 0.90 m depth sensor. In this figure, an interesting phenomenon can be observed. The soil water retentive characteristic causes soil moisture at 0.60 m depth to be higher than at 0.30 m. Without the PISys, the user can only see the surface moisture, but has no information about deeper moisture, unless they make an exploratory pit.

**Figure 15:**
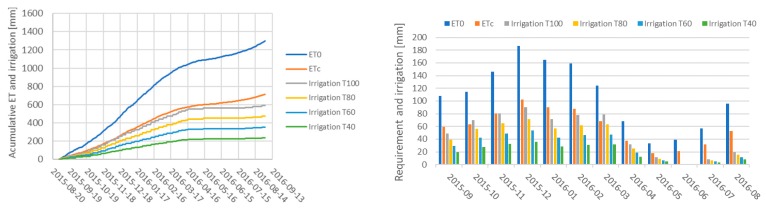
On the left, the cumulative $ET_{0}$, and $ET_{c}$ values are shown, compared to the amount of water applied to B3/U1. Treatment T100 is under PISys monitoring (field station 3) and was always maintained close to the $ET_{c}$. On the right, the amount of water required and that effectively applied are presented. The effect of the RDI treatment during the pit-hardening period can be appreciated in both figures.

The amount of water applied during the experiment, compared to the calculated $ET_{c}$, can be observed in Figure 15 for the four irrigation treatments applied. Although the frequency of irrigation was subject to the characteristics of the irrigation equipment (five irrigation shifts), the PISys was able to satisfactorily adjust the amount of water applied, according to the crop water demand, as can be observed in Figure 15. The total amount of water applied in the $100\%\;ET_{c}$ treatment is similar to that previously obtained by References [54,55].

**Figure 16:**
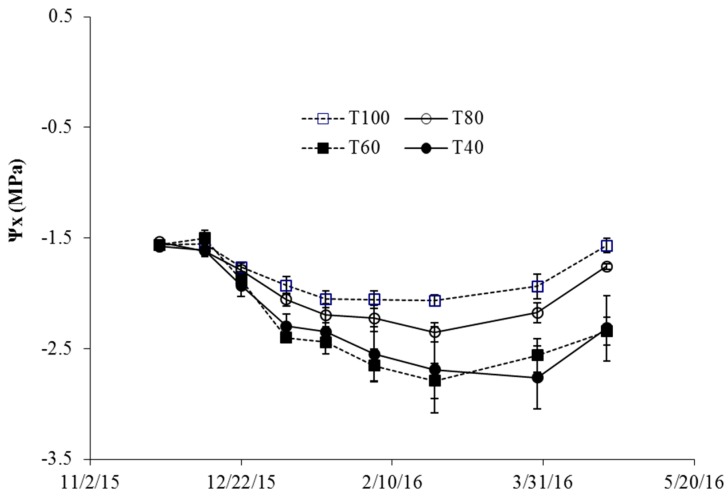
Changes in midday stem water potential ($\Psi_{x}$) in the 2015–2016 seasons. Each point represents the average of the measurements; the vertical bars represent the standard error.

The use of the PISys was useful for ensuring that the irrigation strategies proposed for the different phenological stages were effectively applied. The application of different amounts of water with respect to the treatment under control of the PISys ($ET_{c}$ 100%) allowed the user to know if the irrigation strategy applied could inadvertently generate a situation of water stress in the crop. The evaluation of the SWP ($\Psi_{x}$) throughout the season shows the lack of water stress out of the pit-hardening period. Only during that period, the $\Psi_{x}$ fell to −2 MPa, Figure 16. The SWP for T100 during the whole season coincided with the response of a $100\%ET_{C}$ treatment, as documented in References [48,55]. In the case of treatments T80, T60, and T40, these are Sustained Deficit Irrigation treatments in which the water applied is less than the $ET_{c}$, so the trees do not recover from the water stress as in T100. 

Water deficit treatments showed a diminishment in the SWP throughout the seasons and differences with the T100. The amount of water applied and the levels of $\Psi_{x}$ were similar to what was observed in some experiments where rainfed treatments were evaluated [52,56]. The differences observed indicate not only that the $ET_{c}$ was correctly estimated, but also that the PISys was able to deliver the calculated amount of water to the crop.

## 5. Conclusions

This work presented the development and implementation of a system for measuring and monitoring soil moisture at some representative irrigation units, incorporating the weather variables for proper management of the irrigation equipment on an olive orchard, in an arid region of Argentina. These concepts have been included in the development of the PISys, as a response to the technological demands from local and regional growers.

The main advantages of the system proposed are the remote control and monitoring of the irrigation system, soil moisture and weather variables, and fault detection. These characteristics could result in water and energy savings. The use of the system also makes it possible to implement different controlled irrigation treatments (for example, restriction deficit irrigation). The system can also be adapted to any crop, under any irrigation methodology.

Pilot experiments have shown that the system presented is a useful tool for complementing and assisting with irrigation decisions. The user can program the irrigation schedule based on the crop water requirements, and obtain instant feedback on the irrigation process. Experiments, carried out in a commercial olive orchard, demonstrated that the use of the PISys helps the user to monitor the temporal evolution of $ET_{c}$, the amount of water applied and the soil moisture levels, according to the desired irrigation schedule.

Even though the system was designed with the region’s climate conditions in mind, there are other places around the world where climatic or economic conditions require precise monitoring and control of the irrigation process.

Future works will include comparative results related to the consumption of water, energy, fertilizer, and other resources. Information obtained during the 2014–2015 and 2015–2016 seasons will be used as the basis to analyze water use efficiency (WUE) and the quality of the production.

## Figures and Tables

**Table 1:** Olive orchard and irrigation system details.

Block	Olive Variety	Spacing	Number of Plants	Blok/Unit	Irrigation Valve	Irrigation Shift	Drip Flow	Drippers Separation	Total Drippers	Precipitation Rate	Total Flow
							[L/h]	[m]		[mm/h]	[m^3^/h]
1	Arbequina	6 m × 3 m	4568	1/1	1	1	3.5	1	13,350	1.17	47.9
				1/2	2	4	3.5	1	13,528	1.17	47.5
2	Arbequina	6 m × 3 m	4611	2/1	3	2	3.5	1	14,032	1.17	50
				2/2	4	1	3.5	1	13,380	1.17	46.8
3	Arbequina	6 m × 3 m	4802	3/1	5	5	3.5	1	14,048	1.17	50.3
				3/2	6	1	3.5	1	14,110	1.17	50.2
4	Arbequina	6 m × 3 m	4851	4/1	7	3	3.5	1	14,152	1.17	50.7
				4/2	8	1	3.5	1	14,198	1.17	50.5
5	Coratina	7 m × 3.5 m	3696	5/1	9	5	3.5	0.75	15,216	1.2	54.6
				5/2	10	4	3.5	0.75	14,942	1.2	52
6	Royal Changlot	7 m × 3.5 m	3563	6/1	11	3	3.5	0.75	15,370	1.2	55.1
				6/2	12	2	3.5	0.75	15,068	1.2	52.4
7	Barnea	7 m × 3.5 m	3667	7/1	13	5	3.5	0.75	15,302	1.2	54.7
				7/2	14	4	3.5	0.75	14,038	1.2	49.8
8	Picual	7 m × 3.5 m	3231	8/1	15	3	3.5	0.75	14,786	1.2	53.1
				8/2	16	2	3.5	0.75	14,922	1.2	52.9
9	Barnea	7 m × 3.5 m	1458	9/1	17	4	3.5	0.75	13,842	1.2	48.6
	Koroneiki	7 m × 3.5 m	1429	9/2	18	5	3.5	0.75	13,460	1.2	48.1
10	Frantoio	7 m × 3.5 m	3625	10/1	19	3	3.5	0.75	16,250	1.2	56.8
				10/2	20	2	3.5	0.75	15,932	1.2	55.9
